# Graves’ Disease With Initial Presentation of Thyrotoxic Periodic Paralysis

**DOI:** 10.7759/cureus.49524

**Published:** 2023-11-27

**Authors:** Lloyd Petty, Katie Kaput

**Affiliations:** 1 Endocrinology, Diabetes and Metabolism, University of Utah, Salt Lake City, USA

**Keywords:** symmetric paralysis, hypokalemia, hyperthyroidism, hypokalemic thyrotoxic periodic paralysis, graves´disease

## Abstract

Graves’ disease is a common cause of hyperthyroidism. However, thyrotoxic periodic paralysis (TPP) is a rare complication of Graves’ disease and is characterized by episodes of muscle weakness and hypokalemia in the setting of thyrotoxicosis. Episodic weakness and paralysis can be the first manifestation of Graves’ disease with TPP despite lacking classic symptoms of hyperthyroidism and can be precipitated by risk factors such as a high carbohydrate diet and strenuous exercise. Although TPP is reversible with correction of hypokalemia and thyrotoxicosis, its uncommon presentation can lead to delay in diagnosis and treatment. Here, we describe a case of a 24-year-old Thai male who presented with proximal muscle weakness that progressed to frequent falls and inability to ambulate over the course of three days. He was found to have severe hypokalemia and diagnosed with TPP from underlying Graves’ disease. He was treated with cautious replacement of potassium, a beta blocker, and methimazole to reverse thyrotoxicosis. He regained his ability to ambulate, and his weakness resolved after hypokalemia was corrected. He did not have a reoccurrence of muscle weakness the following 12 months after discharge by continuing treatment with methimazole. The varied clinical manifestations of TPP can make diagnosis challenging, but early recognition and treatment can prevent severe complications of this potentially life-threatening condition.

## Introduction

Thyrotoxic periodic paralysis (TPP) is a rare condition that manifests with hypokalemia and a decrease in muscle strength due to sudden shifts of potassium into cells. It occurs when patients have high circulating levels of thyroid hormone or hyperthyroidism. Graves’ disease is the most common cause of hyperthyroidism associated with TTP [1]. Less frequently it can occur due to other causes of thyrotoxicosis, such as toxic multinodular goiter, iodine-induced thyrotoxicosis, excessive thyroxine use, solitary toxic adenoma, lymphocytic thyroiditis, and amiodarone-induced thyrotoxicosis [1]. In North America, the incidence rate of TPP is approximately 0.1%-0.2% in thyrotoxic patients [2,3]. The incidence in Asian countries is much higher with the incidence rate in Japanese patients with hyperthyroidism at 1.8%, and in Chinese patients with hyperthyroidism at 1.9% [3]. Higher incidence rates have also been recognized in Indian, Thai, Filipino, Vietnamese, and Korean populations [3-5]. However, due to its uncommon occurrence in the United States, this life-threatening, yet reversible condition can easily be overlooked due to its unfamiliar presentation. The following case described a patient who presented with TPP. This article was previously submitted as an abstract at the 2021 ENDO Society Annual Conference on March 30, 2021 [6].

## Case presentation

A 24-year-old Thai male with a past medical history of atrial fibrillation with prior ablation and a strong family history of autoimmune thyroid disease presented to the emergency department with the complaint of acute onset muscle weakness. The patient reported progressive generalized muscle weakness for three days and as his symptoms worsened, he could no longer ambulate. His weakness was associated with frequent falls requiring assistance from others and eventually, he was unable to lift himself from a seated position prompting his friends to bring him to the emergency department. Weakness did not improve with rest. Upon arrival at the emergency department, he was in a wheelchair and was unable to ambulate.

Other notable symptoms included sporadic and non-persistent episodes of tremors and heat intolerance. He also reported having lactose intolerance and loose stools when eating dairy products which was not persistent. The patient otherwise denied unexplained weight loss, palpitations, hair changes, skin changes, or vision changes. He had no associated back pain, numbness, or paresthesia and denied recent fever or viral illnesses.

Family history was remarkable for a mother with Hashimoto’s thyroiditis. No family members were known to have hypokalemic periodic paralysis.

On physical exam, the patient had sinus tachycardia with a heart rate of 120 bpm, temperature was 98.8 degrees F, blood pressure of 140/84 mmHg, and a respiratory rate of 16 bpm. His BMI was 23 (kg/m^2^) with a weight of 68 kg. Physical exam was notable for bilateral 2/5 muscle strength with flexion and extension in the hips, knees, shoulders, and forearms. He also had 2/5 grip strength bilaterally. There was absent patellar and biceps reflex bilaterally and normal sensation to light touch equal on bilateral extremities. No tremors. Mild goiter was present. No proptosis, chemosis or lid lag. No evidence of active thyroid eye disease. No rashes or skin lesions.

Laboratory results were notable for potassium of 1.8 mmol/L (3.3-5.0 mmol/L) and magnesium of 1.8 mg/dL (1.6-2.6 mg/dL). The rest of his chemistry panel, complete blood count, and urinalysis were unremarkable. The patient had athyroid stimulating hormone (TSH) of < 0.01 (0.3-4.00 mU/L), free thyroxine of 5.0 ng/dL (0.8-1.7 ng/dL), total triiodothyronine 301 ng/dL (80-200 ng/dL), and TSH receptor antibody 10.17 IU/L (<=1.75 IU/L). Creatine kinase was 890 U/L (20-200 U/L). MRI of the lumbar spine was obtained and was unremarkable. Electromyography was not available.

The patient was treated with cautious potassium repletion and received 40 mEq oral potassium, 40 mEq IV potassium, and 2 grams of IV magnesium. Serum potassium increased to 4.4 mmol/L and the patient had resolution of his weakness shortly after potassium levels normalized. He did not require further potassium replacement. The patient was also started on propranolol 40 mg BID and methimazole 20 mg daily. At the time of discharge, two days after presenting to the hospital, the patient was able to ambulate independently and had marked improvement in mobility and activity (Table 1).

**Table 1:** Timeline of events TSH; thyroid stimulating hormone, FT4; free thyroxine, TT3; total triiodothyronine, K+; potassium, KCL; potassium chloride, Mg; magnesium, MgSO4; magnesium sulfate.

Timeline	Symptoms	Diagnostic test	Treatment
Initial presentation	Non-ambulatory. Severe proximal muscle weakness 2/5. Heat intolerance. Diarrhea. Tremors.	K+ 1.8 mmol/L	40 meq KCl PO
		Mg 1.8 mg/dL	40 meq KCl IV
		TSH <0.01 mU/L	2 grams MgSO4 IV
		FT4 5.0 ng/dL	Methimazole 20 mg daily
		TT3 301 ng/dL	Propranolol 20 mg BID
+ 7 hours	Weakness improved 4/5 and patient is ambulatory.	K+ 4.4 mmol/L	Monitored in hospital
+ 36 hours	Muscle strength improves 5/5.	K+ 4.2 mmol/L	Discharged from hospital
+ 1 month	Weakness resolved. Mild tremor remains.	FT4 3.1 ng/dL	Increased Methimazole to 30 mg daily
		TT3 203 ng/dL	
+ 3 months	Asymptomatic.	K+ 3.9 mmol/L	Decreased Methimazole to 10 mg daily
		TSH 0.01 mU/L	
		FT4 1.0 ng/dL	Stopped Propranolol
		TT3 91 ng/dL	
+ 12 months	Asymptomatic. No recurrence of muscle weakness.	TSH 10.15 mU/L	Decreased Methimazole to 5 mg daily
		FT4 1.3 ng/dL	

Three months after the initial presentation, the patient continued methimazole, and with improvement in thyroid function tests was tapered down to methimazole 10 mg daily. The patient gained 4 kg in weight, and no longer had symptoms of hyperthyroidism. He did not have a reoccurrence of thyrotoxicosis or episodes of weakness. At follow-up 12 months after the initial presentation, the patient remained on methimazole without reoccurrence, but then moved out of state and was lost to follow-up.

## Discussion

TPP can be identified by thyrotoxicosis, acute hypokalemia, and acute muscle weakness which can progress to paralysis [7]. The periodic episodes of atonia occur after thyrotoxicosis has already developed, and patients may have significant symptoms of hyperthyroidism including palpitations, tremors, unexplained weight loss, heat intolerance, and diaphoresis. Occasionally a precipitating factor can be identified, such as strenuous exercise, eating a high carbohydrate meal, trauma, infections, or acute illnesses. However, the presentation can be variable, and patients can have very subtle or non-classical signs and symptoms of thyroid dysfunction.

When paralysis develops, it is usually symmetric beginning in the lower extremities which can progress to quadriplegia. Bulbar, respiratory, and ocular muscles are typically spared, along with mental function and sensation [4]. The intensity and duration of muscle weakness is variable and can last from a few hours up to several days [4]. The condition can be potentially lethal as paralysis can lead to respiratory failure, and the thyrotoxic state with potassium abnormalities can lead to cardiac arrhythmias and cardiac arrest [8]. Given the potential for mortality, prompt recognition is vital.

In contrast to the typical presentation of Graves’ disease, which occurs more frequently in women between the ages of 40 and 60, TPP is more common in adult males between the ages of 20 to 40 [1, 9]. The prevalence of TPP in men compared to women ranges from 17:1 to 76:1 [3]. On possible explanation for the male predominance in TPP may be related to male androgens affecting the sodium-potassium ATPase pump activity [4].

Studies have proposed that the effects of thyroid hormone (T3) stimulation on the sodium-potassium ATPase activity, or the indirect effect of thyroid hormone on insulin hypersecretion, can play a role in intracellular potassium shifts that lead to episodes of weakness [10]. Other studies have proposed an association of TPP with potential channelopathies due to genetic mutations. Approximately 33% of patients with TPP have a mutation in the KCNJ18 gene, which was found while screening genes for skeletal muscle ion channels in patients with TPP [11]. The gene encodes for the inwardly rectifying potassium channel 18, or Kir2.6, and some of these mutations can directly affect currents in the channels, either inherently or by a mechanism induced in response to thyroid hormone. Whether thyroid hormone increases Kir2.6 transcription or alters Kir2.6 activity, the change in current affects the function in excitable tissues, such as skeletal muscle, and may cause a predisposition for the episodic weakness seen during thyrotoxicosis [11]. Other gene mutations encoding for skeletal muscle channels, such as an R83H mutation in the Kv3.4 gene, and R672G mutation in the gene for Nav1.4 have also been associated with TTP [1].

Although patients with TPP have profound hypokalemia, this is largely related to intracellular shifts, and total potassium stores may be normal. If potassium is repleted with exogenous potassium administration too aggressively, the patient can be at risk of rebound hyperkalemia as they recover from thyrotoxicosis. One study comparing patients with TPP who received normal saline only vs intravenous KCl administration at a rate of 10 mmol/hr in normal saline found that recovery time was significantly shorter in the group that received potassium, but rebound hyperkalemia occurred in a greater percentage of patients [12]. It is therefore advisable to replete potassium cautiously in stable patients. High dose oral propranolol (3-4 mg/kg) is another medication that can affect potassium concentrations by suppressing the activity of sodium-potassium ATPase and blocking the potassium shift into cells [13]. Because of this some studies had shown rapid termination of paralysis with high dose propranolol without having to use potassium [13]. Propranolol may also play a role in the prevention of recurrent attacks of periodic paralysis while patients are thyrotoxic by suppressing the activity of sodium-potassium ATPase [4].

Treatment of the underlying hyperthyroidism with the goal to maintain the patient in a euthyroid state will prevent further episodes of periodic paralysis, and can be accomplished with use of antithyroid medications, radioactive iodine (RAI) therapy or thyroidectomy. While awaiting normalization of the thyrotoxic state, patients should be counseled to avoid precipitating factors such as engaging in strenuous exercise and eating high carbohydrate meals as these factors can affect potassium shifts in cells and exacerbate episodes of paralysis. In North America, antithyroid medications and radioiodine ablation are commonly used as definitive therapy for hyperthyroidism, but the exact modality of definitive therapy is greatly influenced by geographical and institutional practices. Ultimately, the optimal treatment should be a shared decision with the physician and the patient based on the risks and benefits of each treatment option.

## Conclusions

TPP is a rare condition that can be difficult to diagnose, but a thorough medical history, physical exam, and biochemical evaluation can lead to the correct identification and successful treatment of TPP. Thyrotoxicosis should remain in the differential when evaluating patients with hypokalemic paralysis, particularly in Asian ethnicities. Treatment goals involve achieving and maintaining a euthyroid state to prevent complications of hyperthyroidism and further episodes of muscle weakness. Special attention should be given when treating hypokalemia to prevent rebound hyperkalemia by using beta blockers and low-dose potassium repletion cautiously if needed. Avoid risk factors while thyrotoxic that can exacerbate episodes of weakness such as strenuous exercise or high carbohydrate meals.

## References

[1] Kung AW (2006). Clinical review: thyrotoxic periodic paralysis: a diagnostic challenge. J Clin Endocrinol Metab.

[2] Kelley DE, Gharib H, Kennedy FP, Duda RJ, McManis MB (1989). Thyrotoxic periodic paralysis. Report of 10 cases and review of electromyographic findings. Arch Intern Med.

[3] Ober KP (1992). Thyrotoxic periodic paralysis in the United States. Report of 7 cases and review of the literature. Medicine (Baltimore).

[4] Lin SH (2005). Thyrotoxic periodic paralysis. Mayo Clin Proc.

[5] Aggarwal A, Wadhwa R, Pande A, Sahu M, Kapoor D, Khanna R (2019). Hypokalemic periodic paralysis and spectrum of thyroid disorders: analysis of 7 cases from northern India. Indian J Endocrinol Metab.

[6] Petty L, Kaput K (2021). Hypokalemic periodic paralysis as initial presentation for Graves’ disease [abstract]. Endocr Pract.

[7] Tella SH, Kommalapati A (2015). Thyrotoxic periodic paralysis: an underdiagnosed and under-recognized condition. Cureus.

[8] Fisher J (1982). Thyrotoxic periodic paralysis with ventricular fibrillation. Arch Intern med.

[9] Hussain YS, Hookham JC, Allahabadia A, Balasubramanian SP (2017). Epidemiology, management and outcomes of Graves' disease-real life data. Endocrine.

[10] Cesur M, Ilgin SD, Baskal N, Gullu S (2008). Hypokalemic paralysis is not just a hypokalemic paralysis. Eur J Emerg Med.

[11] Ryan DP, da Silva MR, Soong TW (2010). Mutations in potassium channel Kir2.6 cause susceptibility to thyrotoxic hypokalemic periodic paralysis. Cell.

[12] Lu KC, Hsu YJ, Chiu JS, Hsu YD, Lin SH (2004). Effects of potassium supplementation on the recovery of thyrotoxic periodic paralysis. Am J Emerg Med.

[13] Lin SH, Lin YF (2001). Propranolol rapidly reverses paralysis, hypokalemia, and hypophosphatemia in thyrotoxic periodic paralysis. Am J Kidney Dis.

